# Kaposi's Sarcoma-Associated Herpesvirus ORF45 Interacts with Kinesin-2 Transporting Viral Capsid-Tegument Complexes along Microtubules

**DOI:** 10.1371/journal.ppat.1000332

**Published:** 2009-03-13

**Authors:** Narayanan Sathish, Fan Xiu Zhu, Yan Yuan

**Affiliations:** Department of Microbiology, School of Dental Medicine, University of Pennsylvania, Philadelphia, Pennsylvania, United States of America; Harvard Medical School, United States of America

## Abstract

Open reading frame (ORF) 45 of Kaposi's sarcoma-associated herpesvirus (KSHV) is a tegument protein. A genetic analysis with a null mutant suggested a possible role for this protein in the events leading to viral egress. In this study, ORF45 was found to interact with KIF3A, a kinesin-2 motor protein that transports cargoes along microtubules to cell periphery in a yeast two-hybrid screen. The association was confirmed by both co-immunoprecipitation and immunoflorescence approaches in primary effusion lymphoma cells following virus reactivation. ORF45 principally mediated the docking of entire viral capsid-tegument complexes onto the cargo-binding domain of KIF3A. Microtubules served as the major highways for transportation of these complexes as evidenced by drastically reduced viral titers upon treatment of cells with a microtubule depolymerizer, nocodazole. Confocal microscopic images further revealed close association of viral particles with microtubules. Inhibition of KIF3A–ORF45 interaction either by the use of a headless dominant negative (DN) mutant of KIF3A or through shRNA-mediated silencing of endogenous KIF3A expression noticeably decreased KSHV egress reflecting as appreciable reductions in the release of extracellular virions. Both these approaches, however, failed to impact HSV-1 egress, demonstrating the specificity of KIF3A in KSHV transportation. This study thus reports on transportation of KSHV viral complexes on microtubules by KIF3A, a kinesin motor thus far not implicated in virus transportation. All these findings shed light on the understudied but significant events in the KSHV life cycle, delineating a crucial role of a KSHV tegument protein in cellular transport of viral particles.

## Author Summary

Kaposi's sarcoma–associated herpesvirus (KSHV) is a tumor virus associated with Kaposi's sarcoma (KS) and a spectrum of other lymphomas. These tumor cells are usually latently infected with this virus. The inactive virus in cells can get reactivated, whereupon there is viral DNA replication and viral protein synthesis. Newly synthesized proteins assemble in an orderly fashion to form viral complexes that need to be transported to the cell periphery for release and to further infect fresh cells to maintain the infection. Events that make up this important phase in the viral life cycle, however, have been much less studied. In this study, we show that a KSHV protein called the open reading frame (ORF) 45 anchors newly assembled viruses onto a cellular motor protein, namely KIF3A. These viruses are then transported by KIF3A along microtubules which act as major cellular highways (tracks), allowing for efficient transportation of viral complexes toward the cell periphery. Inhibition of any of these steps resulted in a reduced transport of viral complexes reflecting as reduced viral levels. Thus, this study has helped to delineate crucial events involved in the transportation of newly assembled KSHV virions and provides for attractive viral and cellular targets that could be inhibited to reduce the virus burden.

## Introduction

Kaposi's sarcoma–associated herpesvirus (KSHV), also known as the human herpesvirus 8 (HHV-8), is a human DNA tumor virus [1]. KSHV is etiologically associated with the endothelial neoplasm Kaposi's sarcoma (KS) and with certain lymphoproliferative disorders like primary effusion lymphoma (PEL) and multicentric Castleman's disease (MCD) [2],[3]. KSHV infection of cells by default establishes latency. During this phase, there is expression of only a limited number of the viral (latent) genes essential for maintenance of the viral genome with no production of infectious virions [4]. Disruption of latency results in the reactivation of the virus into the lytic phase, with expression of the entire viral gene panel and production of infectious viral particles [5],[6]. These events thus ensure the propagation and transmission of viruses to uninfected cells serving to maintain the infection [7]. Lytic phase is also essential in sustaining the population of latently infected cells that otherwise would be quickly lost by segregation of latent viral episomes as spindle cells divide [8]. The KSHV lytic phase and constant primary infection of fresh cells is thus crucial for both the viral tumorigenicity and the disease pathogenesis.

With the exception of viral DNA replication and viral gene expression, other events that follow viral reactivation including virus assembly, transportation and egress however have been much less studied in KSHV. To gain more knowledge about these events it thus becomes essential to identify the different virion proteins and recognize their functional roles. For this purpose, we purified extracellular KSHV virions from tetra deconyl phorbol acetate (TPA)-induced BCBL-1 cells (that are latently infected with KSHV) by double gradient ultracentrifugation and identified the component virion proteins by a mass spectrometric analysis. By this approach a total of 24 different virion-associated proteins were identified by a mass spectrometric analysis including five capsid proteins, eight envelope glycoproteins and eleven tegument or putative tegument proteins [9].

Among the different virion proteins, the tegument proteins in KSHV as well as in other related gamma herpesviruses have not been well studied. Current knowledge of these proteins comes mainly from studies on alpha and beta-herpesviruses wherein they contribute to three essential functions in the viral life cycle. First, some serve a regulatory function, modulating the host cellular environment during the immediate-early phase of infection like the virion host shutoff protein (UL41) of HSV-1 that degrades host mRNA and shuts down the host translation program [10]. Second, some play vital roles in transportation of capsids to the nucleus along microtubules following virus entry into the host cell [11]–[13]. Third, tegument proteins also participate in the complex chain of events involving herpesviral assembly and egress including transportation of viral complexes [14],[15]. Based on these evidence, the need arises to investigate the functional roles if any of even the less studied KSHV tegument proteins.

One among these proteins in KSHV is ORF45, also recognized as an immediate-early (IE) protein [16],[17]. A vital role of ORF45 in events leading to viral egress following virus reactivation emanated from two observations. First, we had generated an ORF45-null recombinant KSHV using the bacterial artificial chromosome (BAC) system by inserting a premature stop codon into the ORF45 coding sequence [18]. Upon reconstitution into a 293T cell system followed by induction, this mutant produced a much lower yield of progeny virions compared to the wild-type virus though viral gene expression and DNA replication remained unaffected [18]. This suggested that ORF45 could possibly have a role to play in the stages subsequent to viral DNA replication and viral protein synthesis, mostly involving transportation of viral complexes toward viral egress. The second supporting observation came from a yeast two hybrid (Y2H) screening employed to identify ORF45 interacting cellular partners that revealed ORF45 to interact with cDNAs of KIF3A (in this study).

KIF3A is a subunit of kinesin-2, a microtubule (MT) plus-end-directed motor protein. Kinesin-2 comprises two motor subunits, KIF3A and either KIF3B or KIF3C, and a non-motor subunit, KAP3 (kinesin superfamily-associated protein-3) [19]–[21]. Kinesin-2 molecules are expressed ubiquitously and transport cargoes along microtubules (MTs) from the nucleus to the cell periphery [19],[20],[22]. KIF3A comprises of an N-terminal head (motor) domain that attaches to and migrates along MTs and a C-terminal tail that presumably functions as the cargo-binding domain [20],[23].

In this study we thus explored the role and significance of ORF45–KIF3A interaction in the possible transportation of KSHV viral complexes along microtubules toward egress following reactivation from latency. We found that ORF45 through its interaction with the cargo-binding domain of KIF3A docked the entire viral capsid-tegument complexes onto KIF3A which were subsequently transported along microtubules for viral maturation and egress. This to the best of our knowledge is the first report attributing a definitive role of KIF3A in virus transportation.

## Results

### ORF45 interacts with KIF3A

Earlier studies with an ORF45-null mutant virus revealed that disruption of ORF45 yielded 10-fold lowered titers of progeny virions as compared to the wild-type virus though viral gene expression and DNA replication remained unaffected [18]. This finding suggested a role of ORF45 in virion assembly or egress. To further explore the function of ORF45 in these processes, we attempted to identify cellular proteins interacting with it. By an Y2H screening we identified interferon regulatory factor-7 (IRF-7) as an ORF45-associated protein with ORF45 antagonizing interferon- related anti-viral responses [24]. Upon revisiting the Y2H results, another cellular protein was also identified as an ORF45-binding protein. This was KIF3A, a subunit of kinesin-2 and a microtubule (MT) plus-end-directed motor protein. KIF3A, a 702 amino acid protein contains three domains, head (motor), stalk and tail. Two prey plasmids isolated from the screening contained cDNAs for the C-terminal fragments of KIF3A with both fragments encompassing amino acids 409 to 702.

**Figure 1:**
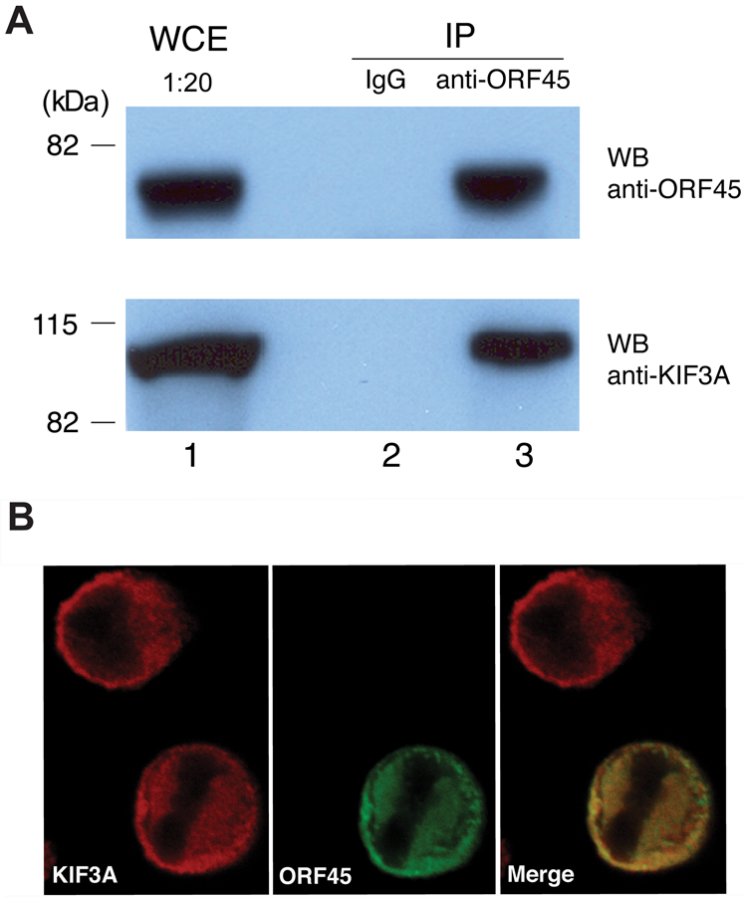
Interaction of KSHV ORF45 with KIF3A. (A) BCBL-1 cells induced with TPA for 48 hours were lysed, and whole cell extracts (WCE) were immunoprecipitated (IP) with mouse IgG (negative control) or mouse monoclonal anti-ORF45 antibody. The immunoprecipitates were analysed by Western blotting (WB) with anti-ORF45 (upper panel) and anti-KIF3A antibodies (lower panel). Positions of the molecular mass standards (kDa) are shown toward the left. (B) BCBL-1 cells treated with TPA for 48 hours were fixed and permeabilized. Cells were subjected to double-labeled IFA using rabbit-polyclonal anti-KIF3A (red) and mouse monoclonal anti-ORF45 (green). The merged image is also shown.

Interaction between ORF45 and KIF3A in cells was further confirmed by co-immunoprecipitation (co-IP) experiments. ORF45 and KIF3A expression vectors were used to co-transfect 293T cells. Forty-eight hours post-transfection, cells were lysed and immunoprecipitated with an anti-ORF45 antibody. By a Western blot, KIF3A was found to be immunoprecipitated with ORF45, suggesting that the two proteins are physically associated in a complex in cells (data not shown). The interaction of these two proteins was also examined in BCBL-1 cells latently infected with KSHV. Cell lysates prepared from TPA induced BCBL-1 cells and immunoprecipitated with a mouse monoclonal anti-ORF45 antibody revealed KIF3A in the immunoprecipitate detected with a rabbit anti-KIF3A antibody (Figure 1A, lane 3). As a control, KIF3A was not immunoprecipitated with mouse IgG (Figure 1A, lane 2). A reverse co-IP performed on cell lysates prepared from TPA-induced BCBL-1 cells with a rabbit-polyclonal anti-KIF3A antibody revealed immunoprecipitation of ORF45 with KIF3A (data not shown). This thus proved the interaction between these two proteins in the reverse direction, too.

Furthermore, the localization of ORF45 and KIF3A in BCBL-1 cells was also examined by immunoflorescence assay (IFA). BCBL-1 cells induced with TPA for 48 hours were fixed and reacted with mouse monoclonal anti-ORF45 and rabbit-polyclonal anti-KIF3A antibodies. Both proteins were exclusively localized in the cytoplasm (Figure 1B). The staining of ORF45 and KIF3A completely overlapped, again suggesting that these proteins are associated with each other in the cytoplasm of the cells.

### Amino and carboxy termini of ORF45 interact with KIF3A

**Figure 2:**
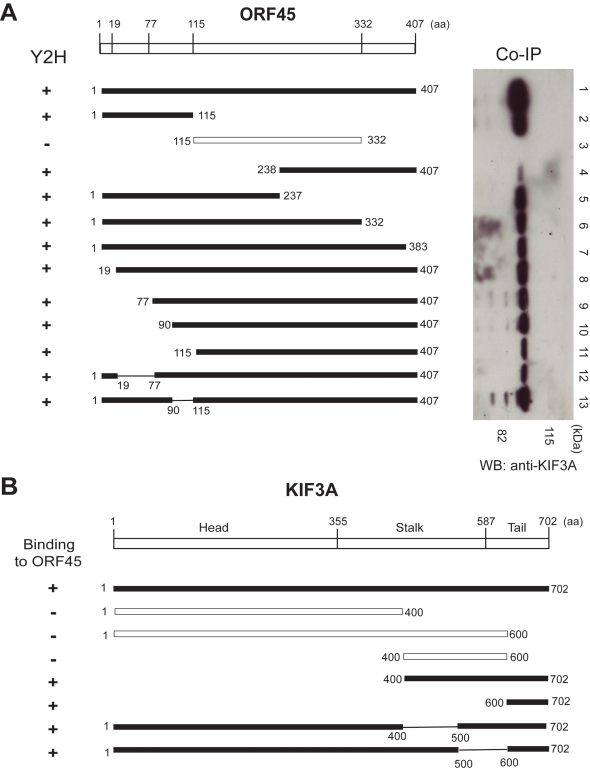
Mapping the domains in ORF45 and KIF3A that are required for their interaction. (A) The domains of ORF45 interacting with KIF3A were mapped by both yeast two-hybrid (Y2H) and co-immunoprecipitation (co-IP) assays. The full-length or the truncation/deletion mutants of ORF45 in either pACT2 (for Y2H) or pCMV-2Flag (for co-IP) vector were used as the prey plasmids. These were tested for interaction with KIF3A bait cloned into pAS2-1 vector (for Y2H) or intrinsically present in 293T cells (for co-IP) by the respective assays. The left panel indicates the Y2H results (β-galactosidase testing performed on yeast colonies cotransformed with the prey and bait plasmids and selected on plates lacking leucine, tryptophan, and histidine with 3-Amino-1,2,4-triazole [3-AT]). The right panel shows co-IP results upon immunoprecipitation of 293T cell lysates (transfected with the ORF45 expression plasmids) with anti-flag affinity gel followed by Western blot (WB) with rabbit-polyclonal anti-KIF3A antibody. Black bars indicate ORF45 prey constructs that interacted with KIF3A, while hollow bars indicate constructs that did not interact with KIF3A. (B) Mapping the domains of KIF3A interacting with ORF45 by an Y2H assay. The prey proteins, either the full-length or the truncation/deletion mutants of KIF3A, were tested against the bait ORF45. Shown on the left are the results of Y2H assay (β-galactosidase testing performed on yeast colonies cotransformed with the prey and bait plasmids and selected on plates lacking leucine, tryptophan, and histidine with 3-AT). Black bars indicate KIF3A prey constructs that interacted with ORF45, while hollow bars indicate constructs that did not interact with ORF45.

To further characterize the interaction between the two proteins, we attempted to map the domains of ORF45 interacting with KIF3A by Y2H and co-IP approaches. For the Y2H assay, full-length and a series of ORF45 truncation/deletion mutants in pACT2 vector (prey) were co-transformed with full-length KIF3A in pAS2-1 vector (bait) into yeast cells. Yeast transformants positive for prey-bait interaction were selected on plates lacking leucine, tryptophan and histidine but containing 3-AT of an appropriate concentration and assayed for β-galactosidase activity. The amino (amino acids 1–115) and carboxy (amino acids 332–407) termini of ORF45 interacted with KIF3A while the central domain (amino acids 115–332) did not interact with KIF3A (Figure 2A, left panel).

For the co-IP assay, a series of ORF45 truncation/deletion mutants were cloned in pCMV–Tag2 vector to express Flag-tagged fragments. The truncation/deletion mutants were introduced into 293T cells by transfection. KIF3A was not included in the transfection as it is ubiquitously expressed in all cells. Upon confirmation of optimal and stable expression of the full-length and the truncation mutants of ORF45 by a Western analysis, cell lysates were immunoprecipitated with anti-Flag M2 affinity gel, followed by a Western blot with an anti-KIF3A antibody. KIF3A was immunoprecipitated with all the truncation mutants of ORF45 from the C-terminus (mutants 1–115, 1–237, 1–332, 1–383) (Figure 2A, right panel, lane 2, lanes 5–7) though with reduced affinities compared to the full-length ORF45 (Figure 2A, right panel, lane 1). A similar finding was also seen with respect to truncations of ORF45 from the N-terminus (mutants 238–407, 19–407, 77–407, 90–407, 115–407) (Figure 2A, right panel, lane 4, lanes 8–11). The central domain (amino acids 115–332) however did not interact with KIF3A (Figure 2A, right panel, lane 3). These findings thus indicated only the amino (1–115) and the carboxy (332–407) terminals of ORF45 to interact with KIF3A, consistent with the Y2H findings.

### The cargo binding domain at the carboxy-terminal tail of KIF3A interacts with ORF45

We next mapped domains of KIF3A interacting with OF45. KIF3A, a 702 amino acid protein consists of an amino terminal head (motor) domain (aa 1–355), a central stalk (aa 356–587) and a cargo binding domain at the carboxy terminal tail (aa 588–702) (Figure 2B). The motor domain has both the ATP and the microtubule binding sites. The tail (cargo binding) domain associates with cargoes transporting them along MTs.

Mapping was performed by an Y2H assay, wherein the prey (KIF3A truncation/deletion segments in pACT2 vector) and the bait (full-length ORF45 in pAS2-1 vector) were co-transformed into yeast cells. Yeast transformants positive for prey-bait interaction were selected as above and assayed for β-galactosidase activity. The KIF3A truncation segments spanning amino acids 1–400 (constituting only the motor domain), 400–600 (constituting only the central stalk) and 1–600 (containing only the motor domain and the stalk) failed to interact with ORF45. However, the segment spanning amino acids 600–702 (constituting the cargo-binding domain) interacted with ORF45. Additionally, deletions of the motor domain (segment 400–702 with Δ 1–399) or the stalk (1–702 with Δ 400–500 or Δ 500–600) did not block the affinity of KIF3A to ORF45. But absence of the cargo-binding domain (1–600 with Δ 601–702) abolished the interaction of KIF3A with ORF45 (Figure 2B). These results suggested that ORF45 specifically interacted with the cargo-binding domain of KIF3A.

### KIF3A associates with entire KSHV capsid-tegument complexes but not with envelope glycoproteins

**Figure 3:**
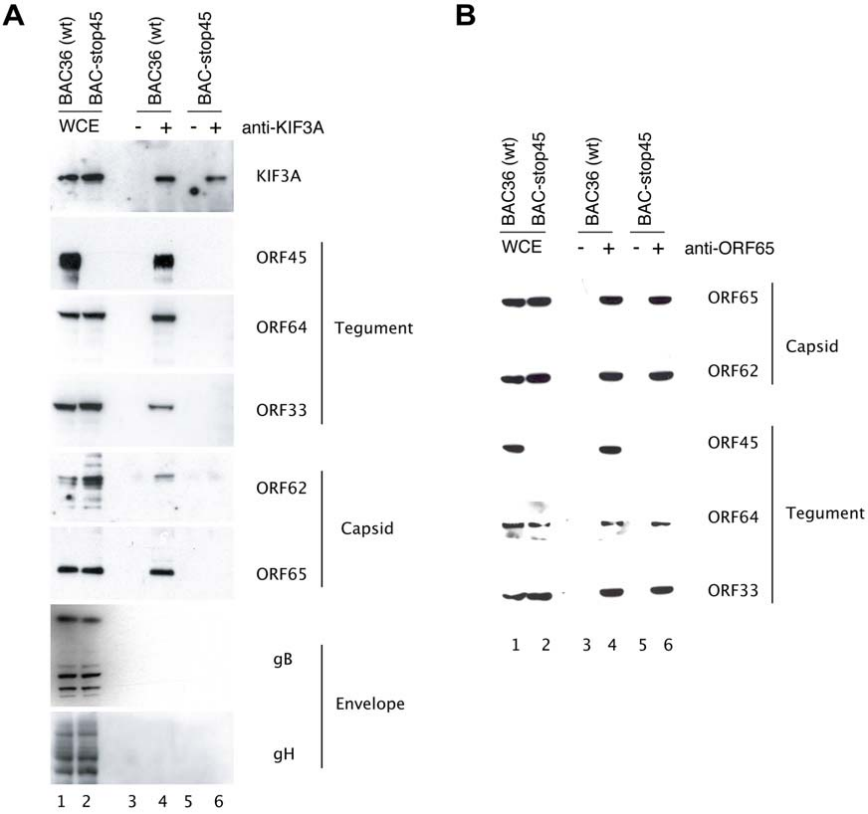
Association of KIF3A with KSHV capsid-tegument complexes mediated by ORF45. Stable 293T monolayers harboring either wild-type (BAC-36) or ORF45-null mutant (BAC-stop45) KSHV were induced with TPA for 48 hours. (A) Whole cell extracts (WCE) from both the BAC36 (lane 1) and the BAC-stop45 mutant (lane 2) were immunoprecipitated with rabbit IgG (lanes 3 and 5) or rabbit-polyclonal anti-KIF3A antibody (lanes 4 and 6). The immunoprecipitates were subsequently analysed by Western blotting with anti-KIF3A and antibodies to the KSHV virion tegument (ORF45, ORF33, and ORF64), capsid (ORF62 and ORF65) proteins, and envelope glycoproteins (gB and gH). (B) The same cell lysates were also immunoprecipitated with anti-ORF65 antibody, and the precipitates were analyzed by Western blotting with antibodies against different capsid and tegument proteins as indicated.

ORF45 specifically binds to the KIF3A subunit of Kinesin-2 and also has been shown to be tightly associated with KSHV tegumented capsids through interaction with many other tegument proteins including ORF64 and ORF63 and capsid proteins ORF 62 [25]. Thus we hypothesized that ORF45 may mediate association of assembling viral particles to the motor molecules and play a role in their transportation along microtubules during virion maturation. To validate the model, we asked if KIF3A associates with only ORF45 or with a whole capsid-tegument complex. We addressed this question by immunoprecipitating cell extracts obtained from TPA induced 293T cells harboring wild-type KSHV (BAC36) with an anti-KIF3A antibody. Upon analyzing the immunoprecipitates, we found that in addition to ORF45, other tegument proteins (ORFs 33 and 64) and capsid proteins (ORFs 62 and 65) were co-precipitated with KIF3A (Figure 3A, lane 4). The viral envelope glycoproteins (gB and gH) however failed to co precipitate with KIF3A (Figure 3A, lane 4). The whole cell extracts (WCE) showed the expression of all the analysed proteins (Figure 3A, lane 1). A similar experiment performed on induced BCBL-1 cells also revealed identical findings (data not shown). Thus these observations suggested that KIF3A is associated and thereby involved in the transportation of only the entire viral capsid-tegument complexes and not the envelope glycoproteins which are transported separately from tegument-capsid particles in the cytoplasm.

### ORF45 mediates the association of KIF3A with the KSHV capsid-tegument complex

We next investigated the specific role of ORF45 in associating viral particles to KIF3A. There were two possibilities (1) ORF45 could just be one among the many KSHV virion proteins interacting with KIF3A; (2) ORF45 probably mediates the association of viral complexes with KIF3A. Hence to delineate the role of ORF45 in this process, we performed a parallel immunoprecipitation assay on cell lysates collected from induced 293T cells carrying an ORF45-null mutant (BAC-stop45). In the absence of expression of ORF45 both the tegument and the capsid proteins failed to coprecipitate with KIF3A (Figure 3A, lane 6), providing a strong evidence that the viral capsid-tegument complexes docked onto KIF3A through ORF45. The lack of co-precipitation of tegument-capsid proteins with KIF3A in the ORF45-null mutant also could have been due to a defect in tegument-capsid complex assembly as a result of lack of functional ORF45 protein. To investigate this posibility, we also performed a co-IP with anti-ORF65 antibody with cell lysates from both BAC36 and BAC-stop45-carrying cells, followed by Western analyses with specific antibodies to some virion proteins. Intact viral capsid-tegument complexes as evidenced by presence of ORFs 65, 62, 33, and 64 were detectable in the immunoprecipitates obtained from both wild-type (Figure 3B, lane 4) and the ORF45-null mutant (Figure 3B, lane 6) viruses. Taken together, these results proved that the absence of functional ORF45 does not disrupt viral tegument-capsid complex assembly processes, but impairs the loading of the viral complexes on kinesin-2 molecules.

### KSHV requires intact microtubules for transport of viral complexes toward egress

**Figure 4:**
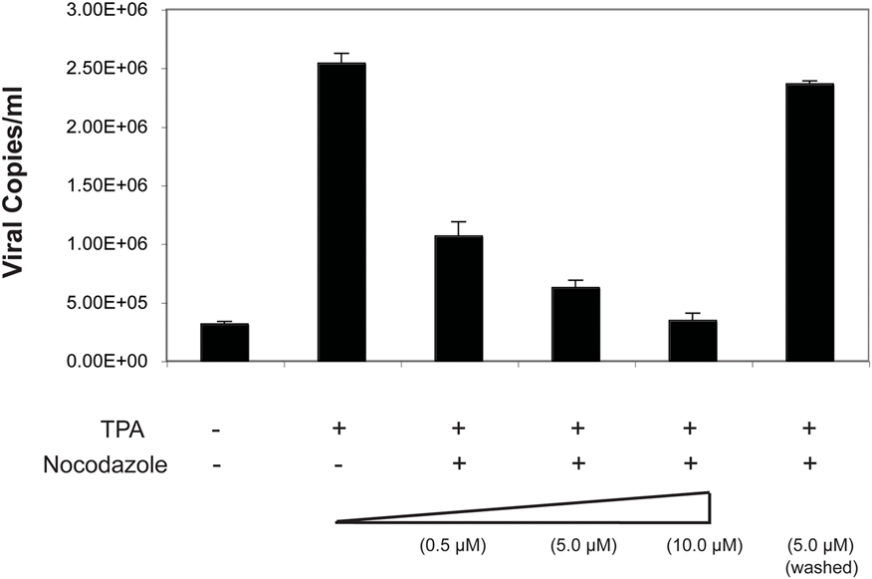
Effect of nocodazole on KSHV virion titers. BCBL-1 cells were treated with increasing concentrations (0.5, 5.0, 10.0 µM) of the microtubule depolymerizing drug nocodazole and induced with TPA. For nocadazole washout experiments, cells were treated with 5 µM of the drug, washed thoroughly to ensure complete removal of the drug, and then induced with TPA. Following induction, extracellular KSHV virions were collected and concentrated 100-fold. Virus stocks were treated with Turbo DNase I and viral DNAs extracted. KSHV genomic DNA concentration was estimated by a real-time PCR along with external standards of known concentrations of the viral DNA using primers against the ORF73 gene and expressed as viral DNA copy numbers per milliliter (ml) of the supernatant.

Studies with other herpesviruses like HSV and pseudorabies virus (PrV) have shown the requirements for an intact MT network to ensure transport of viral complexes toward cell periphery for viral egress [11], [26]–[28]. Additionally, from the association of the KSHV capsid-tegument complexes with KIF3A, a motor protein moving along microtubules, it only seemed logical to investigate the role of MTs in the transportation of the viral complexes following reactivation. To address this issue, BCBL-1 cells were treated with increasing doses (0.5, 5.0, 10.0 µM) of nocodazole, a microtubule depolymerizing drug [29], followed by induction of viral lytic cycle with TPA. Four days post-induction, virions were pelleted and viral copy numbers estimated by a real time PCR. With increasing concentration of nocadazole in a nontoxic range, there was an appreciable decrease in the extracellular virion titers down to a level of 3.5×10^5^ copies/ml amounting to a 8 fold reduction compared to the levels obtained with nocadazole untreated and induced BCBL-1 cells (Figure 4). Hence damage to the MT architecture through depolymerization by nocadazole had contributed to inefficient transport of the viral complexes reflecting as reduced viral titers.

To ensure that the decrease in viral titers seen was not due to the toxic effects of the drug permanently damaging the cellular or the virion architecture, we also performed a nocodazole removal (washout) experiment in parallel. Herein cells treated with 5 µM of the drug were washed twice with PBS and once with serum-free medium to ensure removal of the drug followed by induction with TPA. Such drug removal studies have shown to result in rapid repolymerization of MTs within a short time span [30]–[32]. In our study following the drug removal, a reversal of its effect was evidenced by build up of virion titer levels comparable to that of the untreated but induced cells (Figure 4). Thus drug removal had resulted in MT repolymerization, resuming efficient transport of the viral complexes. To ensure that the decrease in viral titers was not due to nocodazole treatment irreversibly damaging the virion architecture or preventing viral tegument-capsid complex formation, nocodazole-treated and TPA-induced BCBL-1 cells were immunoprecipitated with anti-ORF65 antibody. In addition to ORF65, other capsid (ORF62) and tegument (ORFs 45, 64, and 33) proteins were detected in the immunoprecipitates by Western blot with specific antibodies, indicating the presence of intact tegument-capsid complexes in nocodazole-treated cells (data not shown). This proved that nocodazole did not damage the virion architecture.

**Figure 5:**
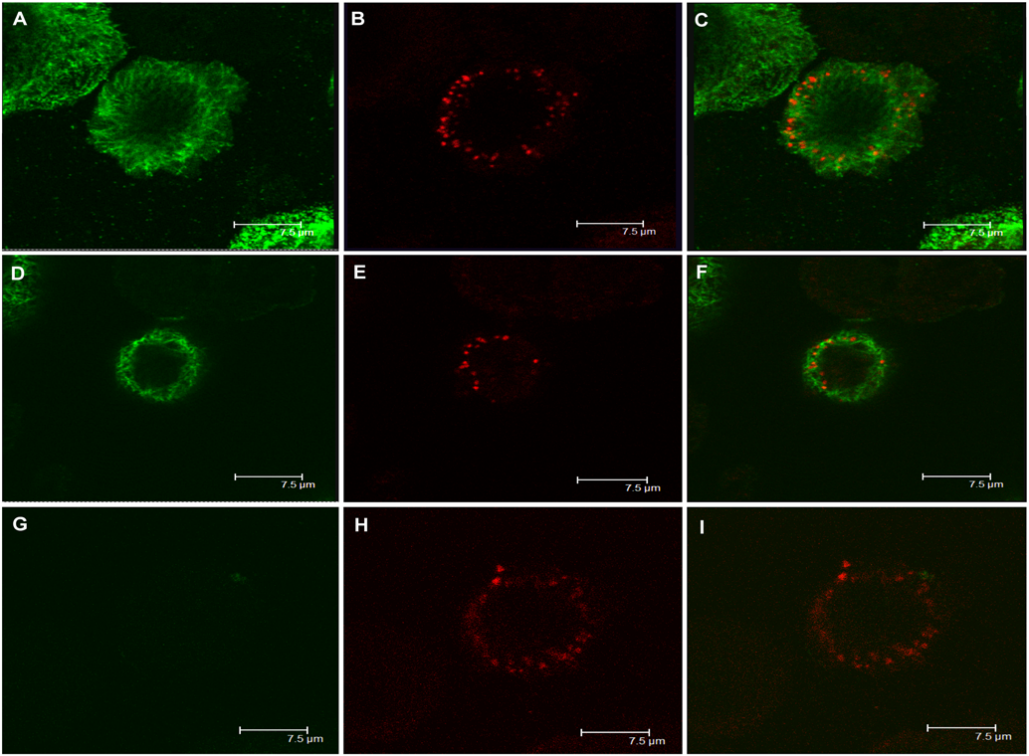
Confocal microscopic examination revealing association of KSHV viral particles with microtubules. BCBL-1 cells induced with TPA for 48 hours were fixed, permeablized, and stained with mouse monoclonal anti-tubulin α and rabbit-polyclonal anti-ORF65 antibodies, respectively. The secondary antibodies were anti-mouse IgG-Alexa Flour 488 (green) and anti-rabbit-IgG-Texas Red (red). The two channels were recorded sequentially and/or simultaneously and images acquired. The individually acquired MT (green channel) (A,D), viral particle (red channel) (B,E), and the merged images (C,F) of a cell are shown. The upper and middle rows represent two different optical sections at which images were acquired from a series of sections collected at 0.32 µm intervals from the bottom to the top of the image. IFA was also performed on BCBL-1 cells that had been treated with 5 µM of nocodazole and induced with TPA (the lower row). The individually acquired MT (G), viral capsid (H), and merged images (I) for a nocodazole-treated cell are shown.

To further investigate if KSHV particles are associated with MTs, a double-labeled IFA was performed. TPA induced BCBL-1 cells were fixed and permeablized. MTs and viral capsids were detected with mouse monoclonal anti-tubulin and rabbit-polyclonal anti-ORF65 antibodies respectively with anti-mouse IgG-Alexa Flour 488 (green) and anti-rabbit–IgG-Texas Red (red) secondary antibodies. Upon confocal microscopic examination, a series of optical sections of the image were collected at 0.32 µm intervals from the bottom to the top of the image. At each interval, the two channels were recorded sequentially and/or simultaneously and images acquired. The individually acquired MT (green channel) and the viral capsid (red channel) staining in a cell are shown in Figure 5A and 5D, and 5B and 5E, respectively, representing two different optical sections at which images were acquired. The merged image shows viral capsids as red structures localized along the MTs (Figure 5C and 5F). A reconstructed 3-D image of the cell constructed revealed close association of viral particles with MTs (Video S1).

IFA was also performed on BCBL-1 cells following nocodazole treatment. Results are shown in Figure 5G–5I and Video S2. First of all, the absence of microtubule network was shown with anti-tubulin antibody staining in the cells treated with nocodazole. Second, we expected to see majority of the viral particles localized only around the perinuclear region. But as shown in Video S2, absence of staining with anti-tubulin prevented us from getting a definitive conclusion. In addition, the cell morphology is another reason for the difficulty to locate the viral particles within the cytoplasm as the nuclear membrane could have been only separated by a few micrometers from the plasma membrane. Thus the viral particles seen could actually have been localized to the perinulear zone, but due to the above phenomenon it might have been difficult to demonstrate it. However, images of the cell taken at different ‘Z’ depths do in fact show viral particles to be more concentrated toward the center of the cell (i.e., the nucleus). Further IFA performed on cells following nocodazole removal gave a picture similar to that of the drug-untreated control with viral particles associated with microtubules (data not shown).

### Functional significance of ORF45–KIF3A interaction

**Figure 6:**
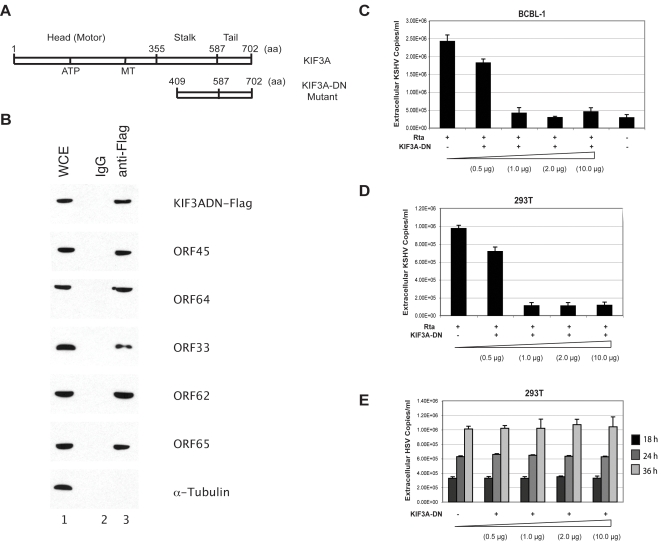
Functional significance of KIF3A-ORF45 interaction. (A) Schematic representation of the wild-type (full-length) and the dominant negative (DN) mutant of KIF3A. (B) Association of KIF3A DN mutant with KSHV tegument-capsid complexes. BCBL-1 cells were electroporated with the KIF3A-DN mutant plasmid along with the RTA-expression vector. Forty-eight hours post-transfection, whole cell extracts (WCE) were immunoprecipitated with mouse IgG (negative control) or anti-flag M2 Affinity gel. The immunoprecipitates were subsequently analysed by Western blotting with antibodies against flag tag, capsid, and α-tubulin proteins as indicated. (C) Increasing concentrations of the KIF3A DN mutant expression plasmid were electroporated into BCBL-1 cells along with 10 µg of pCR3.1-ORF50 or (D) transfected into 293T cells followed by KSHV infection and subsequent TPA induction. Extracellular KSHV virions were collected and concentrated 100-fold. Virus stocks were treated with Turbo DNaseI, viral DNAs extracted, and KSHV genomic DNA concentration estimated by a real-time PCR along with external standards of known concentrations of the viral DNA with primers against the ORF73 gene. Viral titers were expressed as viral DNA copy numbers per milliliter (ml) of the supernatant. (E) 293T cells were transfected with increasing concentrations of the KIF3A DN mutant and infected with HSV-1. Extracellular virions were collected at three different time-points (18, 24, and 36 hours post-infection), and the HSV-1 genomic DNA was extracted and quantitated by a real-time PCR along with external standards of known concentrations of the viral DNA with primers directed against the UL30 gene and expressed as the respective viral DNA copy numbers per ml of the supernatant. Viral titers obtained from the 18, 24, and 36 hour time-point samples are represented as black, light grey, and dark grey bars, respectively.

To investigate the significance of this interaction in the KSHV life cycle, we employed a dominant negative (DN) mutant approach. Such approaches have been often used for functional studies of kinesins, including kinesin-2 [33]–[35]. One among the DN mutants, is a headless mutant, with the head (motor) domain deleted. This mutant is neither able to hydrolyze ATP nor bind to microtubules hence unable to migrate. The KIF3A fragment (amino acids 409–702), isolated from our Y2H screening as interacting with ORF45, could theoretically work as a DN mutant of KIF3A. This fragment is unable to migrate along microtubules as it lacks the N-terminal motor domain (spanning amino acids 1–355, Figure 6A) but could still compete with wild type KIF3A in binding to ORF45 (and thereby viral complexes) due to the intact C-terminal cargo-binding domain.

First, to test if the KIF3A-DN mutant binds to viral particles, BCBL-1 cells were electroporated with the KIF3A-DN mutant plasmid (cloned into a pCMV-flag tagged vector) along with RTA-expression vector (pCR3.1-ORF50 plasmid). Forty-eight hours post-transfection, cell lysates obtained were immunoprecipitated with anti-flag M2 Affinity gel. In addition to ORF45, other virion tegument (ORFs 64, 33) and capsid proteins (ORFs 62, 65) were detected by a Western analysis along with the flag-tagged KIF3A-DN protein. However, the DN protein was not able to bind to microtubules as seen by the absence of alpha-tubulin in the immunoprecipitate (Figure 6B). This suggested that though the DN mutant is bound to viral particles it still cannot transport them along microtubules due to the absence of the N-terminal motor domain.

We then tested if this DN mutant is able to block KSHV particle assembly and release. The KIF3A DN mutant at increasing concentrations (0.5, 1.0, 2.0 and 10.0 µg) was introduced into BCBL-1 cells by electroporation along with pCR3.1-ORF50 plasmid for induction into the lytic phase. Four days post-transfection, extracellular viral particles were collected and quantified by a real-time PCR [18]. With increasing inputs of the KIF3A DN mutant, there was a noticeable decrease in the extracellular virion titers down to a level of 3.7×10^5^ copies/ml, amounting to a 7-fold reduction compared to levels obtained with DN-untreated but -induced BCBL-1 cells (Figure 6C). KIF3A DN mutant thus effectively inhibited the KIF3A-ORF45 interaction indicating that the ORF45-mediated interaction of viral particle to kinesin-2 is crucial for the transport of particles toward viral egress.

To rule out the possibility that the decrease in virion titers seen with the KIF3A DN mutant could possibly have been due to its generalized cytotoxic effects on the cells or other detrimental effects on the viral components, a similar experiment was also performed in 293T cells infected with KSHV or HSV-1. We hypothesized that HSV-1 could serve as an effective negative control based on earlier studies which have documented only the requirements of kinesin-1/kinesin heavy chain (KHC) protein and not that of KIF3A in the transportation of HSV viral complexes toward egress [36]–[38]. Using an RNA interference approach, we also showed that KHC indeed has a significant role in HSV-1 transportation toward egress and that KIF3A plays no discernable role in HSV-1 intracellular transportation (Figure S1). These data clearly illustrated that HSV-1 could be employed as an effective negative control for the KIF3A DN mutant studies.

293T cells transfected with increasing concentrations of the KIF3A DN mutant plasmid (0.5, 1.0, 2.0 and 10.0 µg) were infected with either KSHV (at 50 genomes per cell) or with HSV-1 (5 Pfu/cell). With KSHV, following infection, cells were induced with TPA and virion titers estimated. With increasing inputs of the DN mutant, virion titers dropped to about 1.1×10^5^ copies/ml amounting to a 9-fold decrease compared to levels obtained with DN untreated but induced 293T cells (Figure 6D), similar to that seen with BCBL-1 cells. With HSV-1, at three different time points (18, 24 and 36 hours post-infection), extracellular virions were collected and titers measured by a real-time PCR. There was no noticeable decrease in titers even with increasing concentrations of the KIF3A DN mutant with levels similar to that of the DN untreated control (Figure 6E). Introduction of the KIF3A DN mutant had no detrimental effect on the cells or HSV-1 suggesting that the decrease in viral titers seen with KSHV in both BCBL-1 and 293T cells was specifically attributable to the competitive effect of the KIF3A DN on wild-type KIF3A in binding to ORF45. This clearly points out to the crucial role of KIF3A-ORF45 interaction in pathways leading to KSHV egress.

### Knockdown of KIF3A expression by shRNA-mediated silencing significantly halts KSHV egress

**Figure 7:**
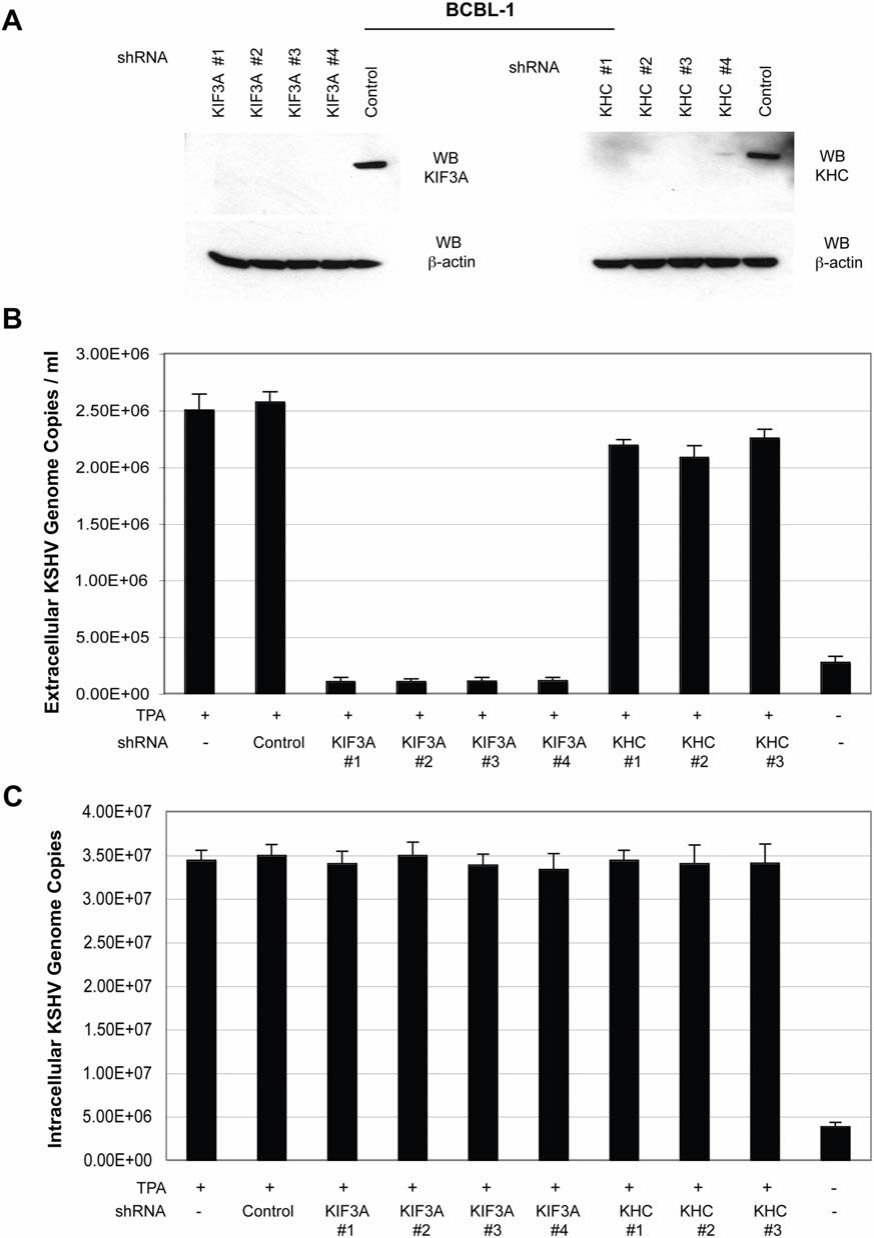
Knockdown of KIF3A and KHC by short-hairpin RNA (shRNA)–mediated silencing and the effects on KSHV egress and extracellular viral titers. (A) Inhibition efficiency of KIF3A and KHC expression in BCBL-1 cells. Two sets of shRNA constructs, each consisting of four individual shRNA lentiviral vectors in pLKO.1-puro plasmids against different target sites of KIF3A (KIF3A shRNA clone #s 1–4) and KHC (KHC shRNA clone #s 1–4), respectively, and a non-targeting control shRNA that activates the RNAi pathway without targeting any known human gene were introduced into BCBL-1 cells through lentiviral transduction in the presence of polybrene. Cells were selected through puromycin (2 µg/ml) selection for a week and then tested for effective knockdown of the respective proteins by a Western blot (WB) performed on the whole cell extracts using anti-KIF3A (rabbit-polyclonal, left panel) and KHC (rabbit-polyclonal, right panel). A WB for β-actin protein detection was also performed as an internal loading control. (B) Effects of KIF3A and KHC silencing on KSHV egress. BCBL-1 cells stably expressing the control shRNA, KIF3A shRNAs (clones #s 1–4), and KHC shRNAs (clone #s 1–3) were treated with TPA (20 ng/ml) to induce KSHV lytic replication. Four days post-induction, extracellular KSHV virions were harvested and concentrated 100-fold. Virus stocks were treated with Turbo DNase I, and viral genomic DNA were extracted. KSHV genomic DNA concentration was estimated by a real-time PCR along with external standards of known concentrations of the viral DNA with primers against the ORF73 gene. Viral titers were presented as viral DNA copy numbers per milliliter (ml) of the supernatant. (C) Effects of lentivirus transduction on KSHV viral DNA replication. BCBL-1 cells stably expressing the KIF3A and KHC shRNAs were treated with TPA (20 ng/ml) to induce KSHV lytic replication. Two days post-induction, cells were collected and total intracellular DNAs were extracted with the Qiagen DNeasy kit. Intracellular viral DNAs were measured by a real-time PCR with primers directed to the ORF73 gene from a 100 ng input DNA. The viral genome copies were normalized to 20,000 copies of GAPDH.

Though the KIF3A DN mutant reduced the KSHV viral egress considerably, we did not notice a complete abrogation of viral egress. There could be two possibilities for this scenario: (i) though greatly reduced by the DN mutant, some full-length KIF3A could still interact with ORF45 mediating viral transportation; (ii) other kinesin motors may also contribute to transportation of viral complexes. To address the first possibility, we attempted to knockdown KIF3A expression in cells through a short-hairpin RNA (shRNA)-based approach and examine its effect on KSHV transportation and hence egress. A Mission shRNA gene set against human KIF3A was purchased from Sigma-Aldrich. The Mission shRNA system is a lentiviral vector based RNA interference library against annotated human genes, which generates siRNAs in cells and mediates gene specific RNA interference for extended periods of time. The KIF3A shRNA set consists of four individual shRNA lentiviral vectors against different target sites of KIF3A mRNA sequence (clone #1 was directed against the 3′ UTR and the clone #s 2–4 targeting the coding sequence). After introduction into BCBL-1 cells by lentiviral transduction, all the four KIF3A shRNA clones were found to effectively down-regulate KIF3A expression to almost undetectable levels in comparison to the control (Figure 7A). BCBL-1 cells stably expressing the KIF3A and control shRNAs, respectively, were induced with TPA. Four days post-induction extracellular virions were collected and titers estimated by a real-time PCR. All of the four KIF3A shRNA clones were effective in drastically reducing the extracellular virion titers down to a level of about 1×10^5^ copies/ml amounting to a nearly 24 - 25 fold reduction compared to the controls (BCBL-1 cells transduced with control shRNA/TPA induced and untransduced BCBL-1 cells/TPA induced) (Figure 7B). The reductions were more pronounced than that with the DN approach.

To ensure that the decrease in viral titers was not due to any deleterious effects of the introduced lentiviruses or the shRNA sequences on KSHV replication, intracellular KSHV genomic DNA were analyzed at forty-eight hours post-induction by a real-time PCR. The viral genome copies were normalized to GAPDH. Levels of viral replication were not significantly altered in the lentivirus transduced cells compared to non-transduced/TPA induced cells (Figure 7C). Hence the decrease in KSHV viral titers represents most likely a direct effect of KIF3A knockdown. Furthermore, this set of KIF3A shRNAs did not affect HSV-1 egress in 293T cells while the HSV-1 production was dramatically inhibited by three shRNAs that target KHC/kinesin-1 (Figure S1). Overall, the findings from the KIF3A knockdown studies clubbed with the conclusions from the DN mutant studies definitively point to a very pivotal role of KIF3A in transportation of KSHV tegumented capsids, establishing the specificity of KIF3A in KSHV transport.

As to the second possibility that other kinesins may also contribute to KSHV intracellular transport, we also included a set of shRNAs against KHC (kinesin-1) in the studies to look into the role of KHC in KSHV transport. KHC is the typified and the most widely studied among the kinesin group of proteins and it has been definitively implicated in transportation of related herpesviruses like HSV-1 [36]–[38] and many other viruses including vaccinia [39] and West-Nile virus structural proteins [40]. Three of four KHC shRNA clones effectively down-regulated KHC expression to almost undetectable levels as compared to the control (Figure 7A and Figure S1A). None of the three KHC shRNA clones exhibited any drastic effects on KSHV egress to that seen with KIF3A but decreasing the viral egress very slightly (just about 1-fold) (Figure 7B). This finding thus does not exclude the utility of KHC in KSHV transport and suggests possibly a very minor role for KSHV egress. Although it is difficult to conclude that kinesin-2 is the only motor molecule necessary and sufficient for KSHV egress as human kinesin family consists of at least 14 different members, our data indicate that KIF3A remains the primary mediator.

## Discussion

An ORF45-null mutant virus was profoundly defective in the release of extracellular mature infectious virions though with no obvious defects in overall gene expression and lytic DNA replication, suggesting a role of ORF45 in virion assembly and egress [18]. Compelling evidences presented in this report substantiate this possible role by revealing the requirement of ORF45 in the kinesin-2-mediated transport of assembled viral particles along microtubules after nuclear egress. The evidences are (i) ORF45 specifically interacts with the cargo-binding domain of KIF3A and is colocalized with the cellular motor protein in the cytoplasm; (ii) Entire viral tegument-capsid complex was associated with KIF3A with ORF45 mediating the association; (iii) Intact microbutules were required for transport of viral complexes toward egress as demonstrated by nocadazole treatment and IFA; (iv) Disruption of KIF3A-ORF45 interaction with a headless DN mutant of KIF3A or through knockdown of endogenous KIF3A expression by siRNAs noticeably decreased KSHV egress reflected as appreciable reduction in the release of extracellular virions; (v) Knockdown of KHC expression had no appreciable effects on the viral egress demonstrating the specificity of KIF3A in KSHV transportation.

**Figure 8:**
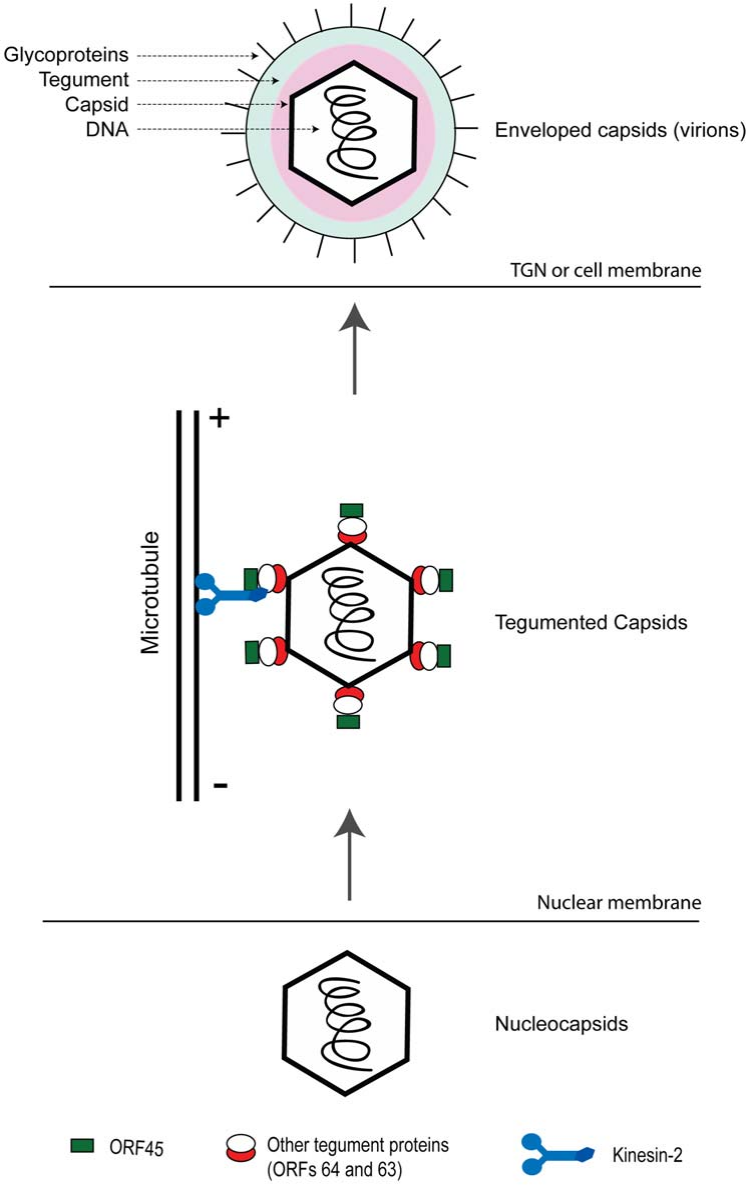
Model for transport of KSHV capsid-tegument complexes following viral reactivation. Newly synthesized nucleocapsids exit out of the nuclear compartment wherein they acquire the tegument proteins including ORF45. ORF45 on the viral particles binds to KIF3A, docking the entire viral capsid-tegument complexes onto it. The complexes are then transported along microtubules either to trans-Golgi vesicles (TGN) or cell membrane for further envelopment and viral egress.

Based on these evidences a model for the role of ORF45 in virion maturation and egress has been presented (Figure 8). In this model, after nuclear egress, KSHV capsids acquire tegument proteins including ORF64, ORF63 and ORF45 in the cytoplasm. ORF45 on the capsid-tegument particles recruits KIF3A and mediates loading of the viral particles onto kinesin-2. Subsequently, viral particles are transported along microtubules in the cytoplasm from the perinuclear region to the cell periphery or trans-golgi network (TGN) membrane where envelopment and egress occur (Figure 8). Transportation and acquirement of envelope glycoproteins is independent to that of the KIF3A transportation of viral tegument-capsid complexes as evidenced in our study wherein KIF3A failed to associate with glycoproteins (gB and gH). Similar findings have also been observed in HSV-1 where the anterograde transport of unenveloped capsids and the glycoproteins in axons involved dissociated pathways [27], [36]–[38],[41].

Similar to ORF45, an identical role of tegument proteins in transportation of viral complexes has been shown in related herpesviruses like HSV-1 and PrV. Specific tegument proteins of HSV-1 like US11 and UL56 interact with related kinesins like the kinesin-1 motors playing vital roles in the anterograde transport of viral complexes along microtubules in the axons [36]–[38],[42]. Outer tegument proteins pUL47, pUL48 and pUL49 of PrV mediate its anterograde transport [11]. The major tegument protein pUL36 in both HSV and PrV contributes to the MT-dependent transport of capsids during egress [26],[28],[43].

Homologues of KSHV ORF45 though found in other members of gamma herpesviruses, are not present in alpha- and beta-herpesviruses. An earlier study from our laboratory revealed a role of ORF45 in antagonizing interferon antiviral defenses. KSHV ORF45 interacted with IRF-7 efficiently suppressing virus-mediated interferon (IFN) gene expression [24]. But the ORF45 counterparts of Epstein-Barr virus (EBV) and Rhesus monkey Rhadinovirus (RRV) did not interact with IRF-7 (our unpublished data), suggesting that interaction with IRF-7 and inhibition of its activation is probably a function unique to KSHV ORF45. In contrast, KIF3A in addition to KSHV ORF45 also interacted with its analogues in EBV and RRV by both Y2H and co-IP (data not shown). The second function of ORF45 in virus transport through interaction with KIF3A could thus be uniformly conserved across all gamma herpesviruses.

An amino acid sequence alignment of ORF45 sequences from related gamma-herpesviruses performed with the ClustalW software (NCBI website) revealed a significant conservation of amino acids in the N-terminal and the C-terminal domains of the protein across the spectrum (data not shown). This observation further substantiated our ORF45 mapping data in KSHV wherein we detected only these highly conserved domains (amino acids 1–115 and 332–407; amino and carboxy terminals respectively) of ORF45 to interact with KIF3A, while the sparsely conserved middle domain (amino acids 115–332) failed to associate with KIF3A.

The C-terminal region (spanning amino acids 600–701) of KIF3A was found to interact with ORF45. This region has been predicted to be the possible cargo-binding domain of the protein [20],[23]. Similar interactions of viral proteins with the cargo-binding domain of other kinesins have also been reported in additional viruses including West-Nile virus [40], HIV-1 [44] and other herpesviruses like HSV-1. Interestingly in HSV-1, US11 tegument protein interacted with the heptad repeat cargo-binding domain of Kinesin-1 [36] contributing to the anterograde transport of nucleocapsids along axons. Interactions of the cargo-binding domain of KIF3A with viral proteins have not been reported thus far. However other cargoes, like the PAR-3 protein (a protein complex functioning in various cell polarization events) [35] and GADD34 (possibly involved in transcription and DNA recombination) [23] specifically interacted with the cargo-binding domain of KIF3A.

To assess if the KIF3A-ORF45 interaction is crucial for the virus in transportation toward viral egress, we further probed their interaction dynamics by two different approaches. The first approach involved the use of a DN mutant of KIF3A that lacks the motor domain but retains the cargo-binding domain. This mutant was seen to compete with full-length KIF3A in binding to ORF45 hence reducing the transport of viral complexes toward viral egress reflecting as a noticeable fall in virion release. Two valid observations arose from this approach: (i) KIF3A–ORF45 interaction is of definitive significance in the transportation of KSHV viral capsid-tegument complexes; (ii) it demonstrates a potential value of the DN mutant in a peptide therapy to treat KSHV-associated diseases which could be further investigated.

With the DN mutant approach, we however did not notice a total abrogation of viral egress. This finding was expected, as the DN mutant can only competitively inhibit the full length KIF3A-ORF45 interaction and not totally abolish the interaction. We hypothesized that a much clearer picture on the contribution of KIF3A in transportation of viral complexes could only arise by approaches that tend to totally inhibit its interaction with ORF45. This was made possible by attempting to knockdown the endogenous KIF3A expression in BCBL-1 cells. A lentivirus based approach was employed to generate cells harboring the KIF3A shRNA sequences integrated into the host cell genome. This allowed for the stable and long-term gene down regulation, overcoming the difficulties associated with only a transient gene knockdown following direct transfection of small interfering RNA (siRNA). Stable knockdown of KIF3A expression manifested as a highly significant reduction in viral egress, more pronounced than that encountered with the DN approach.

Human kinesins constitute a large family of motor proteins with at least 14 members. Our data through gene knockdown studies suggest that kinesin-2 is a major vehicle for KSHV intracellular transport while kinesin-1 (KHC) is crucially responsible for HSV-1 transport. Although the likelihood of other kinesins contributing to KSHV and HSV-1 transport in minor roles cannot be ruled out, the fact that KSHV and HSV-1 egress was clearly not compensated by other kinesins during the shRNA knockdown of KIF3A and KHC respectively exemplifies the specificity of kinesin-2 in KSHV transport and KHC in HSV-1 movement along microtubules.

Though the observations seen with the shRNA mediated knockdown of KIF3A/KHC on KSHV egress, seem to be exciting, these however have to be interpreted cautiously. Down-regulation of the kinesins could possibly have other off-target effects on the viral or cellular components. This could be especially true given the fact that kinesins are involved in cellular processes. Possible deleterious effects on the viral processes associated with the knockdown were ruled out from the observation that the levels of viral DNA replication in cells expressing the KIF3A/KHC shRNAs were similar to that seen in cells expressing the negative control shRNA. More importantly, among the off-target effects on the cells likely to impact on KSHV egress, would be any effects on the microtubule structure. Earlier studies have shown that KIF3A knockdown in mammalian cells do not affect the microtubule architecture [45]. Thus with levels of viral DNA replication and the microtubule architecture remaining unaffected, we could safely conclude that the initial steps in virus assembly remain unaffected by the knockdown. KIF3A down regulation has been shown to produce slightly aberrant Golgi complex morphology in mammalian cells [45]. This is not likely to be the reason for a decrease in KSHV egress, as HSV-1 microtubule-dependent transport was not significantly affected.

The association of KIF3A with the viral capsid-tegument complexes was principally mediated by ORF45. Indeed, ORF45 was found to be tightly associated with the pelleted capsid complex when purified virions treated with detergents (Triton X-100 plus 0.5% DOC) were subjected to high-speed centrifugation [9],[17]. In addition, the interactions of ORF45 with a multitude of tegument proteins including ORF64 and ORF63 and capsid proteins like ORF62 have been revealed in a virion-wide protein interaction study from our laboratory [25]. Thus ORF45 being associated tightly with the capsid-tegument complex is a probable component of the inner tegument acquired early following exit of capsids from the nuclear membrane hence is placed strategically to efficiently position the viral capsid-tegument complexes onto KIF3A. Further mapping studies by Y2H approaches, revealed that ORF45 interacted with other proteins like ORF64 and ORF 63 only through its middle segment (spanning amino acids 115–332) (our unpublished data). This suggested that ORF45 could possibly have 2 distinct interacting domains; the amino and carboxy terminal ends that interact with KIF3A and the central domain interacting with the other virion proteins. This possibly ensures a more efficient binding of KIF3A to its binding sites on ORF45.

In related herpesviruses like HSV-1, recruitment of KHC by tegument proteins contributes to transportation of viral complexes toward viral egress [36]–[38],[42]. Contrastingly our study findings point out that KSHV recruits KIF3A (through ORF45) for transportation of viral capsid-tegument complexes. This finding seems highly intriguing, the most plausible explanation for the differences in the recruited kinesins could reside in the highly divergent tail (cargo-binding) domain of the kinesins that are known to specify distinct set of cargoes for each of them. Given the fact that ORF45, a protein with no homologs in alpha and beta- herpesviruses interacts with KIF3A, it is tempting to speculate that KIF3A might be uniquely adapted to mediate transportation of KSHV and other gamma-herpesviruses.

Two observations in our study substantiated the finding that microtubules serve as the major cellular highway for KIF3A to transport the viral capsid-tegument complexes. Treatment of BCBL-1 cells with a MT depolymerizer nocodazole [29] caused an appreciable decrease in virion production with drug removal replenishing the viral titer levels. Further confocal microscopic images revealed the association of viral particles with MTs following reactivation. Similar requirements of an intact microtubular structure for transport of virus complexes toward egress have been shown for other herpesviruses like HSV and PrV [11], [26]–[28],[38]. Revelation of the process of KSHV particle transport on microtubules during virion assembly provides novel strategies for halting viral replication and treating KSHV-associated diseases. For example, microtubule structure in cells can be disrupted by reagents like nocodazole (an MT destablizer) or taxol (MT stablizer) as many of such reagents are shown to suppress replication of various viruses. Our studies may lead to discovery of new drugs for KS and reveal pharmacological mechanism of the drugs.

In this study we thus report on the transportation of KSHV viral complexes along microtubules by KIF3A, a kinesin motor which to the best of our knowledge has thus far not been implicated in virus transport. This finding could thus lead researchers to investigate similar roles of KIF3A in transportation of other viruses. Furthermore, our study by delineating the role of ORF45 has helped unravel the initial pathways of KSHV transportation toward egress. However further assembly and transportation of mature enveloped virions still remains an enigma. This final transportation phase could probably involve recruitment of kinesin motors either by the glycoptotein cytoplasmic tails or by membrane associated tegument proteins as suggested for HSV-1 [38]. On these lines, a recent study has identified an interaction of HSV-2 membrane-associated tegument protein pUL56 with KIF1A [37]. This interaction has been speculated to contribute to the axonal transport of viral glycoprotein-containing vesicles. Another study has revealed an association of KIF5B with enveloped HSV-1 containing in abundance the amyloid precursor protein (APP), a kinesin cellular receptor [42]. Though these studies provide interesting observations, further investigation of this phenomenon is warranted. The sequence of events involved in transportation of KSHV viral complexes to the nucleus following cell entry also needs investigation with specific focus on the virion proteins and the cellular motors involved. Information garnered from all these sources would definitely help in providing the entire picture of the KSHV lytic life cycle starting from viral entry and ending with viral egress. Lastly, the significance of the less studied KSHV tegument has been exemplified by the pivotal and multi functional roles of ORF45. This should pave way for future studies examining functional roles of even the other tegument proteins in the viral life cycle.

## Materials and Methods

### Cells and viruses

BCBL-1, a latent KSHV-infected primary effusion lymphoma cell line was maintained in RPMI 1640. Human embryonic kidney (HEK) 293T cells were maintained in Dulbecco's modified Eagle medium (DMEM). All cultures were supplemented with 10% heat-inactivated fetal bovine serum (FBS) and antibiotics.

### Plasmid constructs

The prey plasmids used for Y2H were constructed by cloning either the full-length or the different truncation/deletion mutants of KSHV ORF45 and KIF3A (Figure 2A) in pACT2 vector (between the *BamHI & XhoI* sites) in frame with the vector's GAL4 Activation Domain (AD). The deletion mutants of both ORF45 and KIF3A were generated with a PCR-based mutagenesis system, ExSite (Stratagene) using a pair of phosphorylated oligonucleotides in opposite directions with either pACT2-ORF45 or the pACT2-KIF3A as the template plasmids respectively. Bait plasmids were constructed by cloning either the full-length KIF3A or the ORF45 sequences in pAS2-1 vector in frame with GAL4 DNA-binding domain (DBD).

For co-immunoprecipitation (co-IP) assays, full-length and the different truncation mutants of KSHV ORF45 (Figure 2A) were PCR amplified and cloned into pCMV-Tag2 vector (Stratagene) between the *BamHI & XhoI* sites. The deletion mutants of ORF45 were constructed using the ExSite (Stratagene) system with a pair of phosphorylated oligonucleotides using pCMV-Tag2-ORF45 as the template.

### Yeast two-hybrid (Y2H) screen and assays

The ORF45 and KIF3A prey plasmids were tested for their ability to interact with KIF3A and ORF45 bait plasmids respectively by a yeast-two hybrid screen, performed with the Matchmaker system (CLONTECH). The haploid yeast strain MATα MaV103 (gift from Dr. Marc Vidal at the Massachusetts General Hospital) was co-transformed with prey and bait plasmids using lithium acetate. Yeast transformants positive for prey-bait interaction were selected on plates lacking leucine, tryptophan and histidine but containing 3-Amino-1,2,4-triazole (3-AT) and subsequently assayed for β-galactosidase activity using the standard colony-filter assay (as per the Clontech Yeast Protocols handbook).

### DNA transfection

293T cells grown in 100 mm dishes to 70% confluency were transfected with 10 µg of expression plasmid by the calcium phosphate transfection method. BCBL-1 cells were transfected by electroporation. Plasmid DNAs were mixed with BCBL-1 cells in OPTI-MEM medium (Gibco-BRL) and electroporated (200 V, 960 µF) with a Genepulser II (Bio-Rad, Hercules, Calif.). Electroporated cells were then transferred to RPMI 1640 medium supplemented with 10% serum and maintained for the time as indicated.

### Co-immunoprecipitation (co-IP) assay and immunoblotting

BCBL-1 cells were induced with TPA (20 ng/ml) for 48 hours. Cell lysates prepared in ice-cold lysis buffer [25] were homogenized and clarified by high-speed centrifugation at 4°C and subjected to an immunoprecipitation with mouse-monoclonal anti-ORF45 [9] or rabbit-polyclonal anti-KIF3A (Sigma) antibodies for 2 hours or overnight at 4°C. Immunoprecipitated complexes were thoroughly washed with cold lysis buffer, resuspended in 100 µl of sodium dodecyl sulfate-polyacrylamide gel electrophoresis (SDS-PAGE) loading buffer, boiled for 10 mins and loaded onto SDS-PAGE gels (Invitrogen). The primary antibodies used for Western blotting were mouse monoclonal anti-ORF45 and rabbit-polyclonal anti-KIF3A. Subsequent incubation with secondary antibodies and detection was done as earlier [25].

Similarly, transfected 293T cells were collected forty-eight hours post-transfection, lysed with ice-cold lysis buffer and homogenized as above. Immunoprecipitation was performed as above by incubating lysates with anti-flag M2 Affinity gel (A2220, Sigma). Immunoprecipitated protein complexes were washed and run on a SDS-PAGE. Primary antibodies used for Western blotting were the mouse monoclonal anti-Flag M2 antibody (Sigma) and the rabbit-polyclonal anti-KIF3A (Sigma). Incubation with secondary antibodies and subsequent detection was performed as earlier [25].

### Assay of interaction of KIF3A and viral particles using BAC-cloned wild-type KSHV and ORF45-null mutant viruses

Frozen glycerol stocks of the BAC36 (BAC-cloned wild-type KSHV) and BAC-stop45 (ORF45-null mutant virus) [18] were retrieved and BAC DNA was prepared with the large construct kit (QIAGEN). The freshly prepared DNA was transfected into 293T cells, stable BAC-293T monolayers generated through hygromycin selection and induced with TPA. Cells lysates collected forty-eight hours post-induction were incubated with rabbit-polyclonal anti-KIF3A antibody (Sigma) for 2 hours or overnight at 4°C, followed by incubation with Protein G agarose beads (Invitrogen) for 2 hours at 4°C. A reaction with 2 µl of rabbit IgG (Sigma) was included as a negative control. By Western blot, in addition to KIF3A, the IP complexes were also analysed for the different virion protein using specific antibodies including mouse monoclonal anti-ORF45, rabbit-polyclonal anti-ORF64 and mouse polyclonal anti-ORF33 [9], mouse anti-ORF62 IgM (gift from Dr. Z.H. Zhou at UCLA), rabbit-polyclonal anti-ORF65, rabbit-polyclonal anti-gB (gift from Dr. Johnan Kaleeba at the Uniformed Services University of the Health Sciences), mouse monoclonal anti-gH (gifts from Dr. Gary Cohen at Penn).

### Treatment of BCBL-1 cells with cytoskeletal disrupting agent nocodazole

BCBL-1 cells were treated with increasing concentrations (0.5, 5.0, 10.0 µM) of the microtubule depolymerizing drug nocodazole (Sigma) at 37°C for 1 hour and then induced with 20 ng/ml TPA for 4 days. For nocadazole washout experiments, cells were treated with 5 µM of the drug, washed twice with PBS, once with serum-free medium to ensure complete removal of the drug and then induced with TPA. Post-induction, virions were purified and pelleted from the medium supernatant as detailed earlier [9] and were resuspended in 1× phosphate-buffered saline (PBS) in 1/100 of the original volume. Concentrated viruses were first treated with Turbo DNase I (Ambion) at 37°C for 1 h to remove any contaminating DNA outside viral particles. Viral DNA was liberated by digestion with lysis buffer (AL) and proteinase K (supplied with the DNeasy tissue kit, Qiagen) and extracted with phenol-chloroform. Extracted DNA was precipitated with ice-cold ethanol, and the final DNA pellet was dissolved in TE buffer. Copy numbers of KSHV genomic DNA were estimated by real-time DNA PCR with a Roche LightCycler and the LightCycler FastStart DNA Master^Plus^ SYBR green kit with primers directed to LANA [46]. Viral DNA copy numbers were calculated from external standards of known concentrations of BAC36 DNA. A serial dilution of a known amount of BAC36 DNA was used to construct a standard curve. Copy numbers were normalized and were expressed as copy number per milliliter of supernatant.

### Immunofluorescence assay (IFA)

BCBL-1 cells were induced with TPA (20 ng/ml) for 48 hours. Cells were washed with PBS, fixed with 2% paraformaldehyde in PBS, and spun onto Shandon double cytoslides at 1,000 rpm for 5 min. Cells then were permeabilized in 0.2% Triton X-100 in PBS for 20 min on ice and subjected to a double-labeled IFA with mouse monoclonal anti-ORF45 [9] and rabbit-polyclonal anti-KIF3A (Sigma) antibodies. Fluorescein isothiocyanate (FITC)-conjugated anti-mouse IgG and Texas Red-conjugated anti-rabbit IgG (Vector Laboratories, Inc.) were used as the respective secondary antibodies. Slides were examined with a Leica TCS SPII confocal laser scanning system. The 2 channels were recorded simultaneously and/or sequentially and controlled for possible breakthrough between the green and red channels.

For visualization of the viral particles along MTs, induced, fixed and permeablized BCBL-1 cells were subjected to a double labeled IFA. MTs were stained with mouse monoclonal anti-tubulin antibody (Sigma) whilst the viral particles were stained with rabbit-polyclonal anti-ORF65 (capsid antibody). Goat anti-mouse IgG-Alexa Flour 488 (Molecular Probes, Invitrogen) and goat anti-rabbit–IgG-Texas Red (Vector Laboratories) were used as the respective secondary antibodies. Double staining was then examined under a confocal microscope (Leica TCS SPII confocal laser scanning system) by the use of the 495- and 590-nm bands of laser lines from a water-cooled argon-krypton laser under an oil immersion objective. Channel recording and control of data acquisition was done as earlier. A series of optical sections of the image were collected at increasing intervals of 0.32 µm from the bottom to the top. This was employed for the reconstruction of a 3-D representation of the specimen on the graphics computer with the LCS 3D software (Leica Microsystems). Digital images obtained were cropped and adjusted for contrast with Photoshop.

### Dominant negative mutant assay

The C-terminal region of KIF3A (spanning amino acids 409–702) was PCR amplified and cloned into the pCMV 3-Tag- (flag) vector between *BamHI* and *XhoI* sites. This was designated the KIF3A DN mutant. Increasing concentrations (0.5, 1.0, 2.0 or 10.0 µg) of this mutant plasmid along with 10 µg of pCR3.1-ORF50 [47] or empty pCR3.1 vector were mixed and electroporated into BCBL-1 cells as mentioned earlier and maintained for 4 days. ORF50 encodes for the replication transctivator (RTA) essential for induction of cells into the lytic phase [48].

293T cells were also transfected with increasing concentrations (0.5, 1.0, 2.0, 10.0 µg) of the KIF3A DN plasmid by the calcium phosphate transfection method. Following transfection, cells were infected with concentrated KSHV (at 50 genomes per cell) plus Polybrene (4 µg/ml). Virus inocula was then removed, cells were washed twice and replaced with fresh medium containing FBS. Forty-eight hours following infection monolayers were induced with 20 ng/ml TPA (for 4 days). Extracellular KSHV virions were purified and collected from either induced BCBL-1 or 293T cells. DNA extraction from intact virions and subsequent KSHV genomic DNA quantitation was performed as detailed earlier.

KIF3A DN mutant transfected 293T cells were also infected with HSV-1 (5 Pfu/cell) and incubated at 37°C (for 1 hour). Cells were washed twice and replenished with fresh medium containing FBS. At different time points (18, 24 and 36 hours) following infection, the medium was clarified, extracellular virions were collected and DNA isolated. Copy numbers of HSV-1 genomic DNA was estimated by a real-time PCR with primers directed against the HSV-1 UL30 gene [49]. For external standards, HSV-1 genomic DNA was extracted from the HSV-1 KOS strain (kindly provided by Dr. Gary Cohen at Penn) and quantitated. A serial dilution of a known concentration of this DNA was used to construct a standard curve.

### Knockdown of KIF3 and KHC expression using shRNA-mediated silencing technique

Two Mission shRNA gene sets against human KIF3A and KIF5B/KHC respectively were purchased from Sigma-Aldrich. The KIF3A set consists of four individual shRNA lentiviral vectors in pLKO.1-puro plasmids against different target sites of KIF3A (with Clone IDs NM_007054.4-2737s1c1, -2134s1c1, -781s1c1, -1945s1c1 – for convenience sake referred as KIF3A shRNA clone #s 1, 2, 3, 4). The KIF5B/KHC set contains five clones targeting different sites of KIF5B/KHC (with Clone IDs NM_004521.1-3461s1c1, -1376s1c1, -1904s1c1, -391s1c1, -842s1c1 – referred as KHC shRNA clone #s 1, 2, 3, 4, 5). A control vector (a non-targeting shRNA that activates the RNAi pathway without targeting any known human gene, SHC002) was also purchased (Sigma-Aldrich). Each of the shRNA vectors as well as the control vector was used to prepare lentiviral stocks by cotransfecting 293T cells with the shRNA vector and two packaging vectors (pHR'8.2ΔR and pCMV-VSV-G) at a ratio of 4∶3∶1 respectively. . Three days post-transfection, the culture media that contain shRNA retroviruses were harvested, centrifuged (500×g for 10 min at 4°C), and filtered through a 0.45 µm filter to ensure removal of any non adherent cells.

BCBL-1 cells were transduced with the shRNA encoding lentivirus stocks in the presence of polybrene (8 µg/ml). Transduced cells were selected with puromycin (2 µg/ml) for a week. Efficacies of these shRNAs in knockdown of the respective proteins were assayed by Western blot with specific antibodies. BCBL-1 cells stably expressing the KIF3A/KHC/control shRNAs were treated with TPA (20 ng/ml) to induce KSHV lytic replication. Four days post-induction, extracellular KSHV virions were collected. DNA extraction from intact virions and viral titer estimation were performed as detailed earlier.

Sub-confluent 293T monolayer cells were also transduced with the lentivirus stocks in the presence of polybrene and selected with puromycin as above. 293T cells stably expressing the KIF3A/KHC/control shRNAs were infected with HSV-1 at an MOI of 5. Twenty-four hours post-infection, the supernatant medium was clarified, extracellular HSV-1 virions were pelleted and viral DNA isolated. Viral titers were estimated by a real-time PCR as earlier.

## Supporting Information

Figure S1 Knockdown of KIF3A and KHC by short-hairpin RNA (shRNA)-mediated silencing and the effects on HSV-1 egress and extracellular viral titers. (A) Inhibition efficiency of KIF3A and KHC expression in 293T cells. Two sets of hsRNA constructs, each consists of four individual shRNA lentiviral vectors in pLKO.1-puro plasmids against different target sites of KIF3A (KIF3A shRNA clone #s 1–4) and KHC (KHC shRNA clone #s 1–4), respectively, and a non-targeting control shRNA that activates the RNAi pathway without targeting any known human gene were introduced into 293T cells through lentiviral transduction in the presence of polybrene. Cells were selected through puromycin (2 µg/ml) selection for a week and then tested for effective knockdown of the respective proteins by a Western blot (WB) performed on the whole cell extracts using anti- KIF3A (rabbit-polyclonal, left panel) and KHC (rabbit-polyclonal, right panel). A WB for β-actin protein detection was also performed as an internal loading control. (B) Effects of KIF3A and KHC silencing on HSV-1 egress. 293T cells stably expressing the control shRNA, KIF3A shRNAs (clones #s 1–4), and KHC shRNAs (clone #s 1–3) were infected with HSV-1 at MOI of 5. Extracellular virions were collected at 24 hour post-infection. HSV-1 genomic DNA was extracted and quantitated by a real-time PCR along with external standards of known concentrations of the viral DNA with primers directed against UL30 gene and expressed as the respective viral DNA copy numbers per ml of the supernatant.
(1.33 MB TIF)

Video S1 A reconstructed 3-D movie of a cell showing close association of viral particles with microtubules. BCBL-1 cells induced with TPA for 48 hours were fixed and permeablized. MTs and viral capsids were stained with mouse monoclonal anti-tubulin and rabbit-polyclonal anti-ORF65 antibodies, respectively, with anti-mouse IgG-Alexa Flour 488 (green) and anti-rabbit/IgG-Texas Red (red) secondary antibodies. Double staining was then examined under a confocal microscope (Leica TCS SPII confocal laser scanning system). A series of optical sections of the image were collected at increasing intervals of 0.32 µm from the bottom to the top. This was employed for the reconstruction of a 3-D representation of the specimen on the graphics computer with the LCS 3D software (Leica Microsystems).
(1.55 MB MOV)

Video S2 A reconstructed 3-D movie of a cell following nocodazole treatment. BCBL-1 cells treated with 5 µM nocodazole were subsequently induced with TPA for 48 hours, fixed, and permeablized. Microtubules and viral capsids were stained with mouse monoclonal anti-tubulin and rabbit polyclonal anti-ORF65 antibodies, respectively, with anti-mouse IgG-Alexa Flour 488 (green) and anti-rabbit/IgG-Texas Red (red) secondary antibodies. Double staining was then examined under a confocal microscope (Leica TCS SPII confocal laser scanning system). A series of optical sections of the image were collected at increasing intervals of 0.32 µm from the bottom to the top. This was employed for the reconstruction of a 3-D representation of the specimen on the graphics computer with the LCS 3D software (Leica Microsystems).
(1.50 MB MOV)
