# Supplementary figures and images for: Kaposi's Sarcoma-Associated Herpesvirus ORF45 Interacts with Kinesin-2 Transporting Viral Capsid-Tegument Complexes along Microtubules
Source: PLoS Pathog. 2009 Mar 13;5(3):e1000332. doi: 10.1371/journal.ppat.1000332 (PMC2647735; doi:10.1371/journal.ppat.1000332)

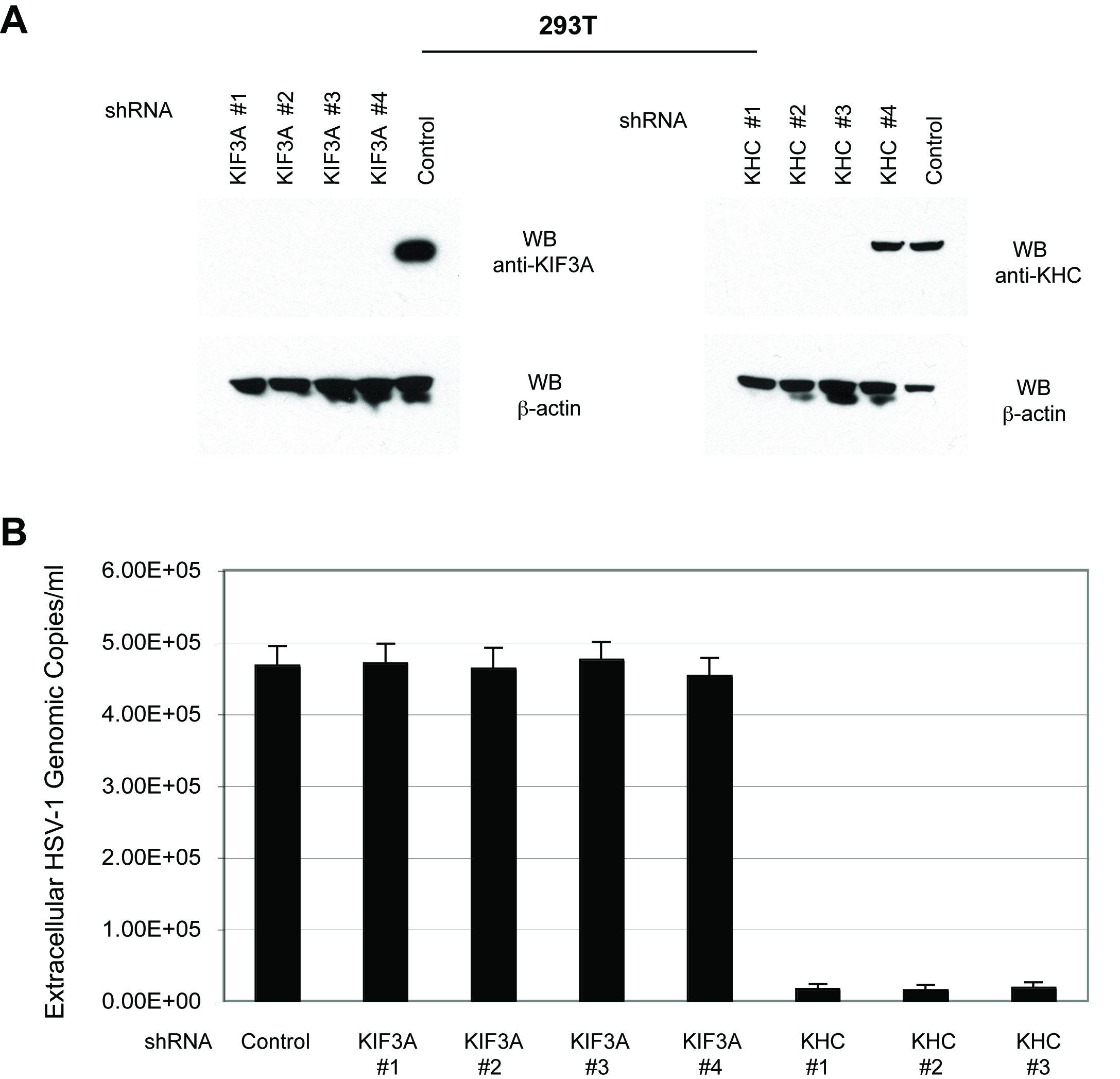

Supplement: Figure S1 — Knockdown of KIF3A and KHC by short-hairpin RNA (shRNA)-mediated silencing and the effects on HSV-1 egress and extracellular viral titers. (A) Inhibition efficiency of KIF3A and KHC expression in 293T cells. Two sets of hsRNA constructs, each consists of four individual shRNA lentiviral vectors in pLKO.1-puro plasmids against different target sites of KIF3A (KIF3A shRNA clone #s 1–4) and KHC (KHC shRNA clone #s 1–4), respectively, and a non-targeting control shRNA that activates the RNAi pathway without targeting any known human gene were introduced into 293T cells through lentiviral transduction in the presence of polybrene. Cells were selected through puromycin (2 µg/ml) selection for a week and then tested for effective knockdown of the respective proteins by a Western blot (WB) performed on the whole cell extracts using anti- KIF3A (rabbit-polyclonal, left panel) and KHC (rabbit-polyclonal, right panel). A WB for β-actin protein detection was also performed as an internal loading control. (B) Effects of KIF3A and KHC silencing on HSV-1 egress. 293T cells stably expressing the control shRNA, KIF3A shRNAs (clones #s 1–4), and KHC shRNAs (clone #s 1–3) were infected with HSV-1 at MOI of 5. Extracellular virions were collected at 24 hour post-infection. HSV-1 genomic DNA was extracted and quantitated by a real-time PCR along with external standards of known concentrations of the viral DNA with primers directed against UL30 gene and expressed as the respective viral DNA copy numbers per ml of the supernatant. (1.33 MB TIF) [file ppat.1000332.s001.tif]
